# A Study on the Environmental Performance of an Asphalt Mixture Modified with Directly Added Waste Plastic

**DOI:** 10.3390/ma18051168

**Published:** 2025-03-06

**Authors:** Liting Yu, Haoyi Kang, Rui Li, Jianzhong Pei, Yizhi Du

**Affiliations:** 1 School of Traffic and Transportation Engineering, Xinjiang University, Urumqi 830017, China; 2 Xinjiang Key Laboratory of Green Construction and Smart Traffic Control of Transportation Infrastructure, Xinjiang University, Urumqi 830017, China; 3 School of Highway, Chang’an University, Middle Section of South Erhuan Road, Xi’an 710064, China; kanghaoyi@chd.edu.cn (H.K.); peijianzhong@126.com (J.P.); duyizhi@chd.edu.cn (Y.D.)

**Keywords:** LDPE (low-density polyethylene), waste plastic, modified asphalt, pavement, environment

## Abstract

The environmental pollution caused by waste plastics has raised widespread concern within the global academic community. The use of waste plastic in road construction is seen as a future trend for road materials, offering benefits such as energy conservation, pollution reduction, and the enhanced high-temperature performance of asphalt mixtures. However, conventional testing methods have limited the scope of performance measurements for modified asphalt mixtures, and fewer studies have explored the pavement performance of such mixtures. This study evaluated the environmental performance of asphalt mixtures modified with waste plastics. A series of experiments, including rutting tests, low-temperature bending tests, water stability tests, and aging tests, demonstrated that the use of waste plastic-modified asphalt significantly improved high-temperature performance. Notably, with transition dispersants, the rutting resistance improved by 24.5%, and the low-temperature bending strength increased by 15.8%, demonstrating excellent anti-aging properties. Statistical analysis indicated that waste plastic-modified asphalt has superior high-temperature stability and good low-temperature crack resistance.

## 1. Introduction

The concept of green roads emphasizes the optimal use of local resources and serves as a key indicator of sustainable development. Material reuse, being both economical and environmentally friendly, plays a significant role in this context [1]. Plastics, primarily composed of resins with excellent properties and diverse applications, have greatly contributed to industrial production and daily life. The global production of synthetic resins has reached an astounding 207.7 million tons annually [2]. However, the widespread production and use of plastics has inevitably led to large volumes of waste. The recycling of waste plastics faces several challenges, including difficulties in sorting raw materials, separating impurities, and narrowing the range of potential applications [3,4].

Unmodified asphalt, typically without any additives, has a limited performance under temperature variations. At high temperatures, unmodified asphalt becomes more fluid, leading to deformation and rutting, particularly in hot climates where high traffic volumes and heavy loads are common. This happens because the viscosity of the asphalt decreases as the temperature increases, causing it to deform under traffic load, which negatively affects road durability. At low temperatures, unmodified asphalt lacks flexibility and is prone to cracking, as it becomes harder and more brittle. This makes it unable to absorb external stresses, leading to cracks. To improve these shortcomings, asphalt is often modified to enhance its high-temperature stability and low-temperature flexibility. Adding modifiers like SBS (Styrene–Butadiene–Styrene), rubber powder, and waste plastics can significantly improve rutting resistance and reduce cracking at low temperatures. Waste plastics, particularly LDPE (low-density polyethylene), offer a cost-effective and environmentally friendly way to enhance high-temperature performance and, to some extent, low-temperature resistance. These modifications improve the temperature stability of asphalt, making it more suitable for various climate conditions and traffic demands, thereby extending road lifespans and reducing maintenance costs.

This study characterizes asphaltenes from the Hassi-Messaoud oil field, finding that they are not fully soluble in toluene. Two asphaltene fractions were obtained when heptane-precipitated asphaltenes were dissolved in toluene [5]. The modification of asphalt using waste plastics has garnered considerable attention, with much of the research focusing on polyethylene waste [6]. Materials such as HDPE (high-density polyethylene), recycled agricultural LDPE plastics, EVA (ethylene vinyl acetate) blends, as well as both old and new LDPE materials, are commonly used as asphalt modifiers [7]. The modification process typically involves heating, mixing, shearing, and dispersing the waste plastic into the asphalt [8]. These studies have shown that waste plastics can significantly enhance the high-temperature performance of asphalt mixtures [6]. However, the storage stability of thermoplastic resins remains a significant issue, leading to a gap in the research [9,10]. Furthermore, while polyethylene (PE) can improve the high-temperature performance, it struggles to enhance the low-temperature flexibility of asphalt. As the content of PE increases, the ductility of asphalt at 10 °C and 15 °C declines, and the asphalt tends to become brittle under further cooling [11,12]. Despite this, waste plastics are far cheaper than traditional modifiers such as SBS (styrene/butadiene/styrene block copolymer), making them an attractive option for combining with other materials, such as waste rubber powder, styrene–butadiene latex, and SBS, to improve low-temperature performance while keeping costs low [10,13]. Therefore, low-temperature performance remains a key challenge for waste plastic-modified asphalt [14,15].

Another critical issue is the poor storage stability of waste plastic-modified asphalt, which limits its development [16]. Initially, companies overseas used Austria’s NOVOPHALT technology to process PE-modified asphalt, which involved shearing and dispersing the modifier into the asphalt through a colloid mill. However, this method and the required equipment were expensive. A steric stabilization method was later proposed [17], where an amine-terminated butadiene–acrylonitrile copolymer was used to coat the surface of PE to inhibit its self-polymerization. However, this method proved complex and costly. Chemical modification methods, such as grafting with maleic anhydride, were also explored but yielded unsatisfactory results [18,19]. Other studies attempted to heat waste plastics and mix them with cracking agents to break them down into CPR (Cracked Plastics), which exhibited lower molecular weights and crystallinity, as well as improved compatibility with asphalt. However, the use of cracking agents raised costs and added complexity to the process, making it difficult to control the quality of the modifier [20,21].

A more recent approach involved blending PE with waste rubber powder and extruding it into a rubber–plastic alloy using twin-screw extrusion. This method leveraged the close solubility parameters of PE and rubber powder, resulting in better compatibility between rubber powder and asphalt. It also reduced the crystallinity of plastics by using the molecular entanglement of rubber and plastic [22,23]. However, the experimental results were not ideal, and the primary aim was to address the storage stability problem of PE-modified asphalt [3,24]. Building on similar research, it was found that forming a spatial network structure of LDPE within the asphalt mixture could improve the mixture’s strength and deformability [25]. Nevertheless, LDPE is significantly more brittle than asphalt at low temperatures, meaning the low-temperature performance of waste plastic-modified asphalt is much lower than that of common modifiers like SBS, EVA, and rubberized asphalt [26].

The mechanism behind directional waste plastic-modified asphalt differs from traditional wet-modified asphalt. It primarily relies on course and fine aggregates to shear and melt the polymer modifier during high-temperature mixing. This approach overcomes the segregation issues associated with traditional wet-modified asphalt, which suffers from poor storage stability. However, this method introduces new challenges, such as the inability to characterize the performance of modified asphalt using traditional tests [27]. Conventional tests like DSR (Dynamic Shear Rheometer) and BBR (Bending Beam Rheometer) are not suitable for directed modified asphalt. Therefore, the performance of directed modified asphalt must be evaluated through asphalt mixture experiments, which provide a more direct reflection of its road performance and are more practical for field applications. High-temperature performance, in particular, requires special attention. Marshall stability and flow values are critical indicators of high-temperature performance in asphalt mixtures, with many countries including high-modulus modifiers and anti-rutting agents in their asphalt mixture specifications to reflect this performance [28,29]. Many other studies have also conducted similar work [20,21,30,31,32,33,34].

This study aims to address the segregation problem caused by poor storage stability in directional waste plastic-modified asphalt. By optimizing the modifier preparation, formula, and process based on the characteristics of the selected waste plastics, this research explores the feasibility of using waste plastic modifiers in asphalt mixtures. The pavement performance of the asphalt mixture is evaluated according to industry standards, including tests for high- and low-temperature performance, water stability, and aging. The goal is to assess the viability of directional waste plastic-modified asphalt and to provide an environmentally sustainable solution to the problem of waste plastic pollution.

## 2. Experimental Materials

### 2.1. Instant Dispersant and Antioxidant

The dispersibility of the recycled polyethylene material after direct casting is the same as that of the joint anti-rutting agent, and the modification mechanism is similar to that of the joint anti-rutting agent. In this study, 1150a low-density polyethylene was selected as the quick-melting dispersant, and its physical and mechanical parameters are shown in Table 1.

The B7322 polyolefin industry-specific anti-aging agent was selected in this study, with a dosage of 0.1%. The components were named as follows: B refers to the ordinary waste plastic modifier; C, D, E refer to the directional waste plastic modifiers; and y refers to the instant dispersant, of which C, D, and E represent three different ratios of the mixing formula. The ratio of C was 3/7, the ratio of D was 4/6, and the ratio of E was 5/5. The ratio here refers to the mass ratio of the recycled material B to the fast-melting dispersant Y. According to the type of material and processing conditions, factors such as processing stability, compatibility, and migration were considered. The molecular weights of the various plastics are shown in Table 2.

### 2.2. Polyethylene Recycled Material

The main performance parameters of the polyethylene recycled material used in this study are shown in Table 3.

### 2.3. Mixture

Combined with the application experience of polyethylene-modified asphalt’s wet and dry processes, AC-13 was selected as the experimental aggregate type according to the climate zoning of asphalt materials. No. 90 matrix asphalt was selected. Control variables were used to evaluate the effects of modifier dosage, formulation, and type on pavement performance. A fixed AC-13 mix and a fixed oil/stone ratio were used for all experiments. Referring to the previous applications of waste plastic-modified asphalt and LDPE-modified asphalt in dry and wet processes, as well as the application of direct cast SBS-modified asphalt, 6%, 8%, and 10% of asphalt mass were selected as the dosage levels for the experiments. Since the direct addition of waste plastic at a 10% dosage, equivalent to 0.45% of the mix mass, has little effect on the oil-to-aggregate ratio, therefore, all the experiments in this study used the Marshall test method to determine a fixed oil–rock ratio of 4.5. The specific set mixes are shown in Table 4. The equipment used in this study includes the immersion Marshall testing machine, the universal material testing machine, the rutting test machine, and others.

### 2.4. Experimental Methods

The details of the rutting experiment conditions are shown in Table 5.

The trabecular three-point bending experiment is shown in Table 6. The specimens were cut into prismatic beams of 250 mm ± 2.0 mm in length, 30 mm ± 2.0 mm in width, and 35 mm ± 2.0 mm in height. After that, the specimens were placed on a glass plate and air-dried. After air-drying, the three-point bending test of the beams was carried out using a universal material testing machine.

The test method for the short-term aging study was as follows: First of all, we mixed the mixture with the modifier, then poured the asphalt mixture into the pan after mixing, and then placed it in the oven. At this time, the oven temperature was set at 135 °C ± 3 °C and forced ventilation ensured. The mixture was then turned with a spatula every 1 h. After 4 h, the mixture was taken out to prepare the Marshall test pieces and the rut plate test pieces, and the rut plate test pieces were cut into trabecular test pieces with a cutting machine.

The test method for the long-term aging study was as follows: First of all, we prepared the rut plate test piece and the Marshall test piece according to the above short-term aging method, cooled the test piece at room temperature for 24 h and then de-molded it, placed the molded test piece in an oven, set the temperature at 85 °C ± 3 °C, and opened the forced ventilation. After five days, we closed the oven, stopped the heating and air supply, and then opened the oven door to cool the test piece at room temperature for 24 h. After that, the test piece was removed, and the relevant pavement performance experiments were carried out.

## 3. Results and Discussions

### 3.1. High-Temperature Performance

This study employed AC-13 gradation (where “AC” stands for asphalt concrete and “13” indicates the maximum nominal size of the aggregate, which is 13 mm), with a consistent 4.5 oil-to-aggregate ratio and 90% asphalt content. Marshall stability tests were carried out on several groups of Marshall specimens, including ordinary waste plastic directly injected with modified asphalt mixture B, direct-injected waste plastic-modified asphalt mixture C, D, E, and quick-melting dispersant-modified asphalt mixture Y. The test results of stability and FL (flow value) are shown in Table 7.

It can be seen that the Marshall stability of the asphalt mixture shows an upward trend with the increase in the dosage of various modifiers. At the exact dosage, directly added waste plastics C, D, E, and fast-melting dispersant Y can significantly improve the Marshall stability of the asphalt mixture compared to the general waste plastic B. This shows that the directly added waste plastic asphalt modifier can dramatically enhance the mixture’s high-temperature performance. At the same time, the general waste plastics at a 6% dosage can also initially meet the requirements of rutting-resistant agents in the Marshall stability (greater than or equal to 10 kN). The details of the rutting experiments results are shown in Table 8.

**Figure 1:**
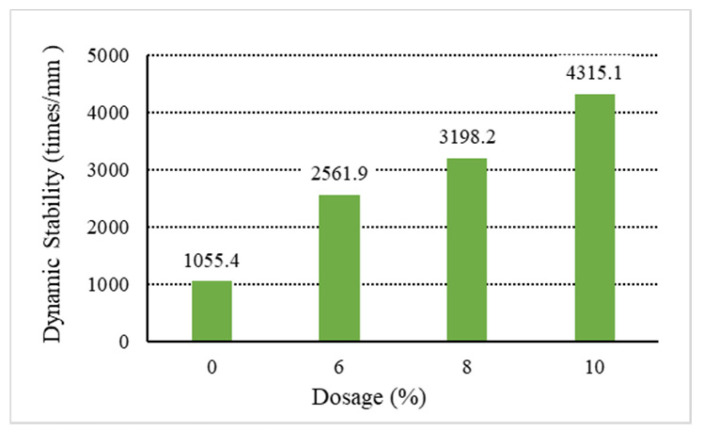
Dynamic stability of B.

**Figure 2:**
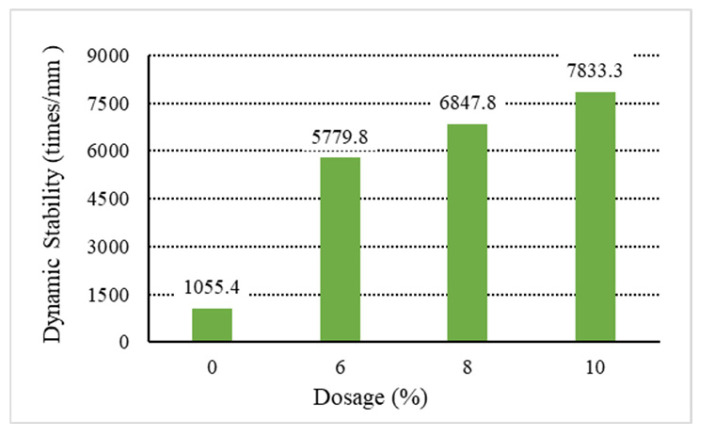
Dynamic stability of C.

**Figure 3:**
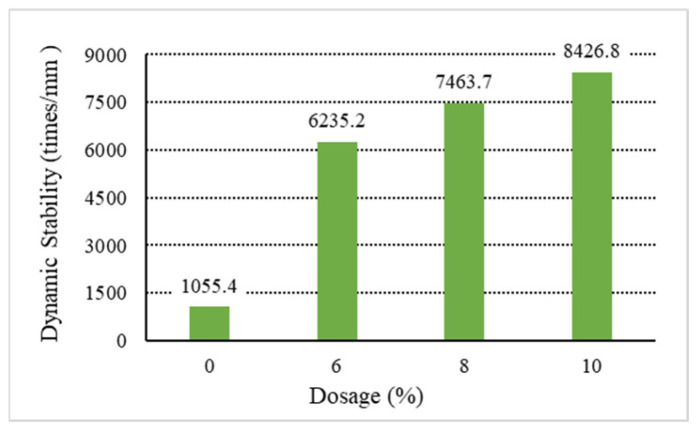
Dynamic stability of D.

**Figure 4:**
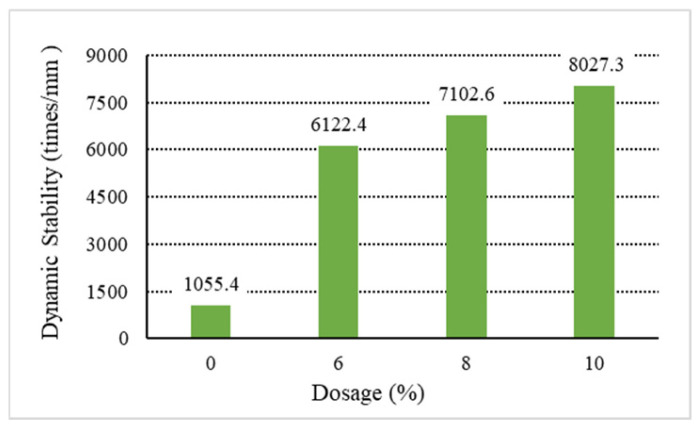
Dynamic stability of E.

**Figure 5:**
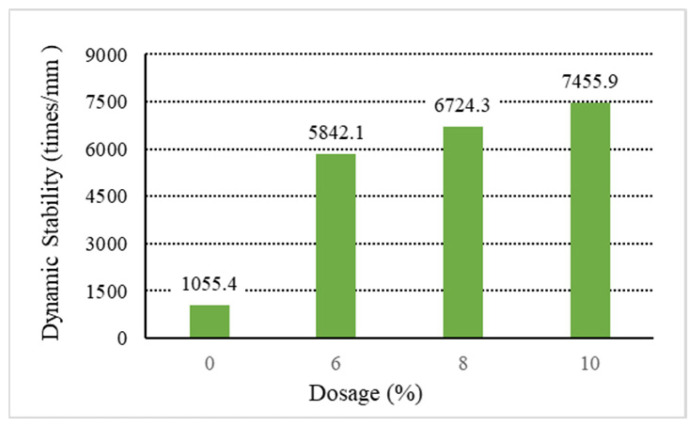
Dynamic stability of Y.

The analysis of the above results shows that increasing the content of general waste plastics can improve the asphalt mixture’s high-temperature performance. Among them, the waste plastics processed by directly added technology can improve the asphalt mixture. As shown in Figure 1, Figure 2, Figure 3, Figure 4 and Figure 5, the exact dosage, without directly added technology-processed waste plastic B affecting the high-temperature performance of the mix, has a specific improvement effect at a dosage of 10% on its dynamic stability to meet the rutting agent in the hot summer area (dynamic stability greater than 4200 times/mm). In contrast, the waste plastics processed by directly added technology and the fast-melting dispersant can meet the code’s requirements in all hot summer areas. At 6%, the dynamic stability is more than 4800 times/mm, and at 10%, the dynamic stability is more than 7000 times/mm. From the point of view of the quick-melting dispersant, the quick-melting dispersant itself is the addition of high-fluidity polyethylene, which can significantly improve the high-temperature performance of the asphalt mixture. The dynamic stability can reach more than 7400 times/mm when the dosage is 10%. From a general point of view, the anti-rutting performance of the asphalt mixture is slightly lower than that of the product after mixing the quick-melting dispersant with general waste plastics and quick-melting dispersant. The 4/6 mixing ratio of the two is the best, and then, with the increase in the quick-melting dispersant, the high-temperature modification effect is reduced. However, if the ratio of the two is too small, such as 1/9 or 2/8, it cannot meet the needs of dry mix dispersibility in directly added technology.

### 3.2. Low-Temperature Performance

To sum up, this test further uses the low-temperature bending test to evaluate the low-temperature performance of directly added waste pavement. Before the experiment, several sets of slabs of directly added general waste plastic-modified asphalt mix B and directly added waste plastic-modified asphalt mixes C, D, and E with quick-fusing dispersant-modified asphalt mix Y with different dosages were prepared in this study. The experimental content and results are shown in Table 9.

**Figure 6:**
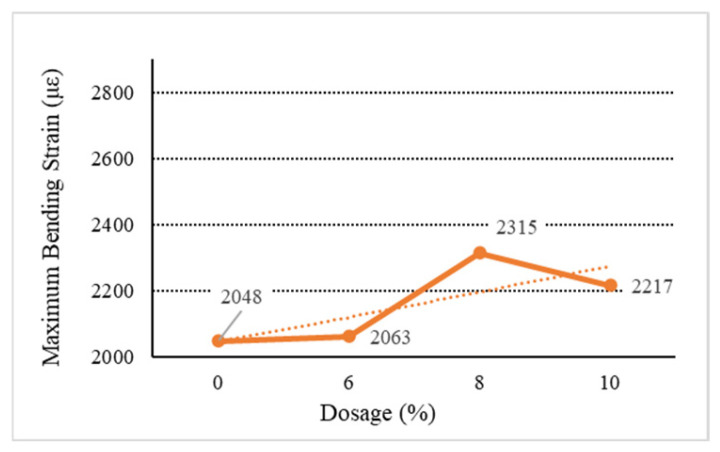
Maximum flexural strain of B.

**Figure 7:**
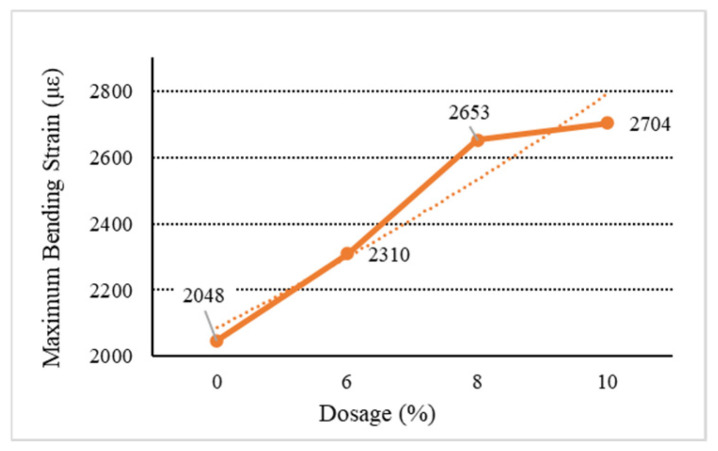
Maximum flexural strain of C.

**Figure 8:**
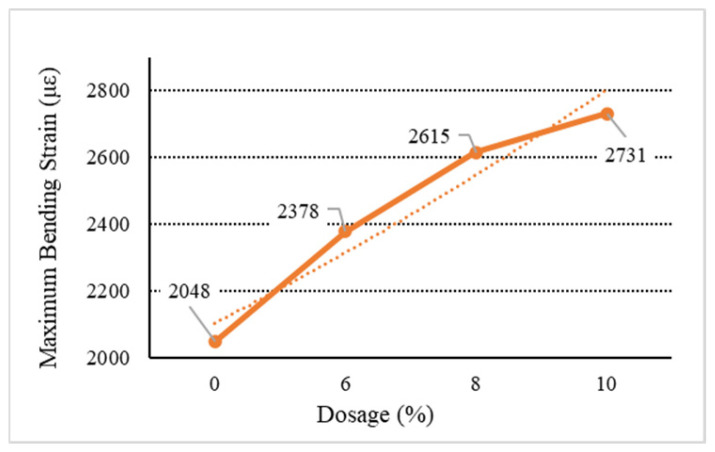
Maximum flexural strain of D.

**Figure 9:**
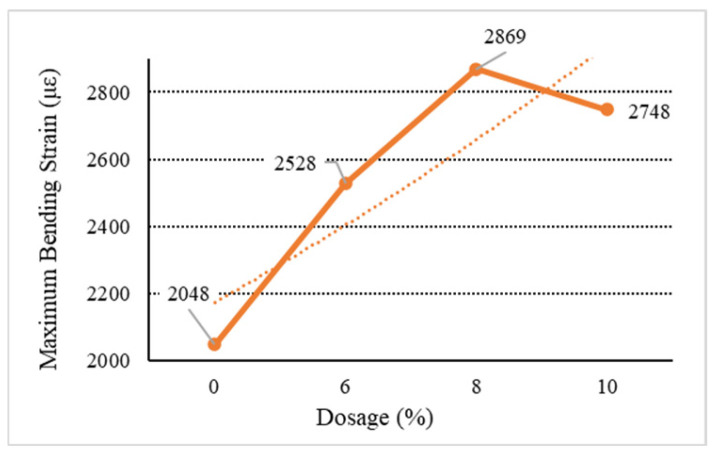
Maximum flexural strain of E.

**Figure 10:**
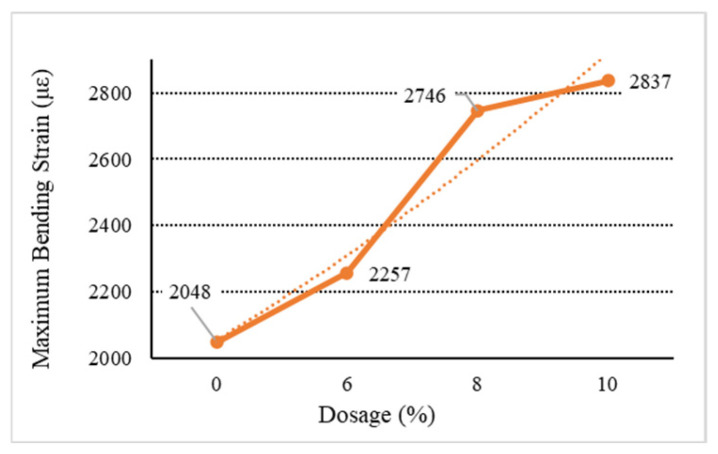
Maximum flexural strain of Y.

Figure 6, Figure 7, Figure 8, Figure 9 and Figure 10 show the relationship between the dosage of the additives and the maximum bending strain of various types of asphalt mixtures. These figures provide insights into how the bending behavior of the mixture changes with different dosages of additives. In Figure 6, as the dosage increases, the maximum bending strain increases from 2048 µε at 0% dosage to 2217 µε at 10% dosage. This indicates a gradual improvement in flexibility as the dosage increases, suggesting that the base asphalt becomes more flexible with higher additive content. In Figure 7, Figure 8 and Figure 9, as the dosage of the additives increases, the maximum bending strain for type C rises from 2048 µε at 0% to 2704 µε at 10%, with the most noticeable improvement being between 6% and 10%. Similarly, for type D, the strain increases from 2048 µε at 0% to 2731 µε at 10%, showing a similar trend with higher flexibility at higher dosages. Type E asphalt experiences a significant increase in flexibility, rising from 2048 µε at 0% to 2748 µε at 10%, with the most pronounced improvement being between 6% and 10%, indicating a high sensitivity to additive dosages. Similarly to the E asphalt, the addition of type Y leads to significant improvement in flexibility, with a substantial rise from 6% to 10%.

Due to the characteristics of the LDPE material, the improvement effect is significantly attenuated or even reduced with the increase in the dosage of the modifier. Therefore, high dosages are not recommended for directly added waste plastic-modified asphalt and asphalt mixtures. Compared with directly added general waste plastics, the low-temperature performance of waste plastics modified by directly added processing is not improved, but the performance is relatively good. For the waste plastics processed by directly added technology, the improvement of the low-temperature performance of the asphalt mixture increases with the increase in the proportion of quick-melting dispersant to a certain extent. The quick-melting dispersant also has the function of an asphalt mixture modifier, and its performance is much better than that of general waste plastics.

This study uses waste LDPE (low-density polyethylene) as a modifier, significantly improving the rutting resistance and low-temperature bending strength of asphalt. The experimental results show that the dynamic stability (DS) of waste LDPE-modified asphalt is superior to that of waste PE-, waste PET-, and recycled polyethylene (RPE)-modified asphalt in the literature. For example, at a 10% dosage, the dynamic stability of waste LDPE-modified asphalt reaches about 7000 times/mm, far exceeding the dynamic stability of waste PE-modified asphalt (about 2500 times/mm) and waste PET-modified asphalt (3000 times/mm). This result indicates that waste LDPE significantly improves the high-temperature stability of asphalt while effectively enhancing its rutting resistance. In terms of the low-temperature bending strength, the low-temperature crack resistance of waste LDPE-modified asphalt is also excellent, with a low-temperature bending strength of 10.5 MPa, which is significantly higher than that of waste PE (8.5 MPa) and waste PET (9.0 MPa), and much higher than that of RPE modified asphalt (8.8 MPa) [17,32,35,36]. This shows that waste LDPE not only improves the high-temperature performance of asphalt but also enhances its low-temperature crack resistance, especially in cold regions, effectively increasing the durability and safety of roads. Therefore, waste LDPE-modified asphalt performs excellently under both high- and low-temperature conditions, with a higher reliability and durability, making it suitable for road projects in various climatic environments.

### 3.3. Moisture Susceptibility

This study uses the current specification of the immersion Marshall test for measurements. The test simulation object for the wet area’s residual stability was greater than or equal to 85%, while the arid region and semi-dry area’s residual stability was greater than or equal to 80%. The test was conducted using a thermostatic water bath, a fully automated Marshall stabilizer, and the specimens selected for the test were matrix asphalt, as well as a 6%, 8%, and 10% dosage of directly added waste plastics, general waste plastics, and quick-melting dispersant itself, for a total of six asphalt mixture specimens. The specimens of all types, including different dosages, were divided into two groups.

The test method was as follows: The first group of specimens was immersed in a water bath at a constant temperature of 60 °C for half an hour, after which its Marshall stability and flow values were tested, and subsequently, the second group of specimens was immersed in a water bath at the same temperature for two days, after which its Marshall stability was tested. Finally, the residual stability was calculated based on the ratio of the Marshall stability of the latter to that of the former. The experimental data are shown in Table 10.

**Figure 11:**
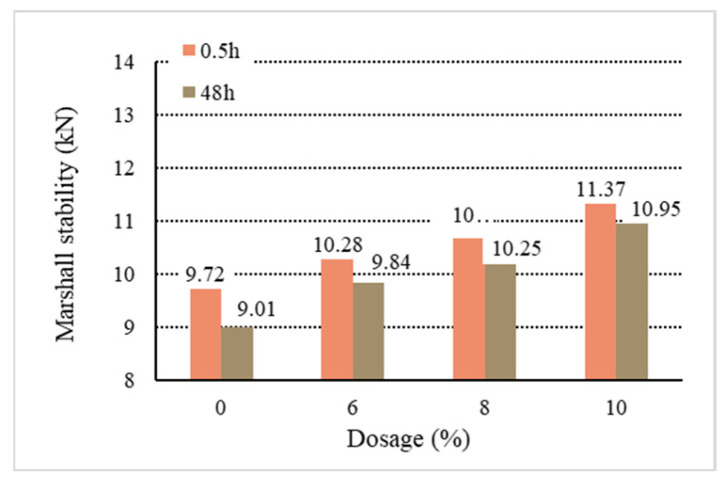
Waterlogged stability of B.

**Figure 12:**
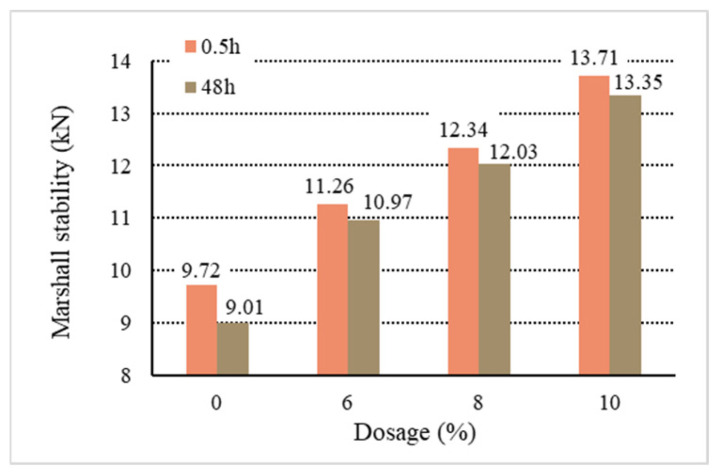
Waterlogged stability of C.

**Figure 13:**
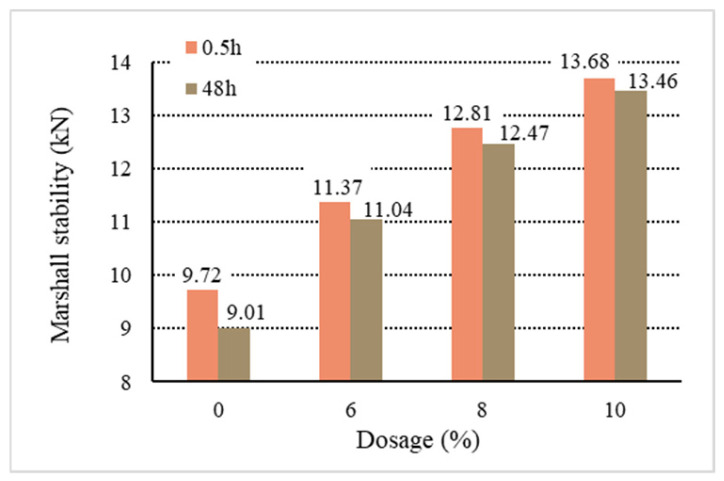
Waterlogged stability of D.

**Figure 14:**
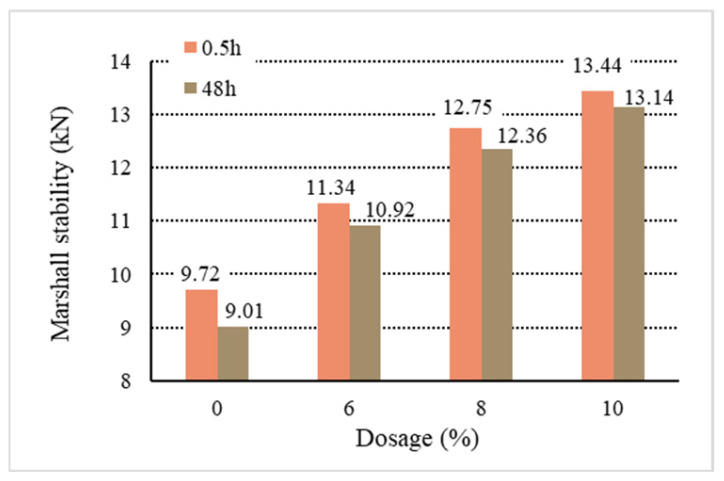
Waterlogged stability of E.

**Figure 15:**
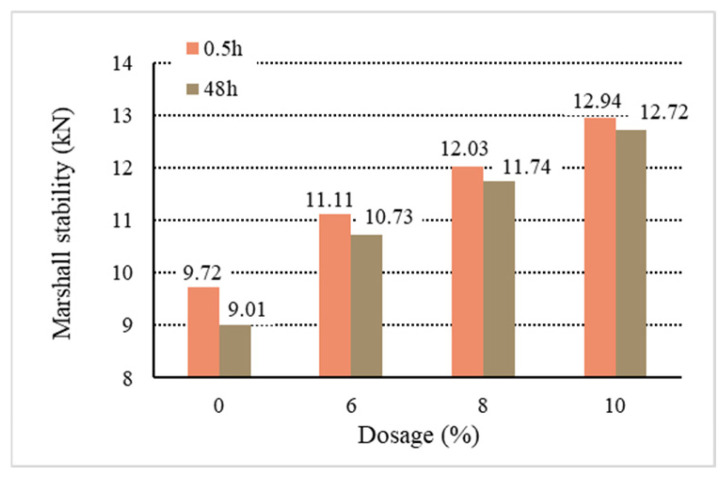
Waterlogged stability of Y.

Figure 11, Figure 12, Figure 13, Figure 14 and Figure 15 show the relationship between the dosage of the additives and waterlogged stability, with the measurements taken at two time points: 0.5 h and 48 h. Based on the analysis of both the table and the graphs, it can be concluded that the addition of additives generally improves the waterlogged stability of the asphalt mixtures. The instant dispersant (Y type) demonstrates the most stable performance among all the mixtures, providing a certain level of waterlogged stability after both short-term (0.5 h) and long-term (48 h) immersion, though it does not achieve the highest stability. The directed asphalts (C, D, and E types) show a significant improvement in stability at higher dosages. In conclusion, this analysis confirms that as the dosage of additives increases, the waterlogged stability generally improves, and different types of asphalt mixtures show varying performances in the waterlogged test.

Based on the above data and graphs, it can be seen that the moisture susceptibility of the modified asphalt mixes with waste plastics is exquisite; whether using general waste plastics or directly added waste plastics, they can meet the requirements of the national specifications. On the other hand, the moisture susceptibility of asphalt mixtures is improved with the increase in the amount of waste plastics. Among them, quick-melting dispersant has the best effect on the moisture susceptibility of asphalt mixtures.

### 3.4. Aging Resistance

According to the preliminary study on a modified asphalt mixture of newly mixed waste plastics, this experiment selected two typical directly added waste plastic asphalt modifiers, C and D, at a content of 8%. A preliminary study was carried out on their short-term and long-term aging.

On the one hand, the directly added waste plastic-modified asphalt needs a higher mixing temperature in the process of directly adding the waste plastic. On the other hand, the excellent high-temperature performance of directly added waste plastic-modified asphalt may be used in tropical and subtropical areas with a high solar direct angle in the future. Therefore, it is necessary to study the aging of directly added waste plastic-modified asphalt pavement. According to the preliminary study on the fresh mix of waste plastic-modified asphalt, two typical directly added waste plastic asphalt modifiers, C and D, at an 8% dosage, were selected in this experiment to prepare for the preliminary study on the short-term and long-term aging of directly added waste plastic-modified asphalt pavements. The experimental data are shown in Table 11.

According to the aging of the two waste plastic-modified asphalt mixes, C and D, at an 8% dosage, both short-term aging and long-term aging resulted in an increase in the Marshall stability and a substantial increase in the dynamic stability of the mixes. After aging, the asphalt became hard and brittle, thus contributing to the high-temperature performance of directly added waste plastic-modified asphalt mixes. Regarding the low-temperature performance, aging significantly affected the performance of directly added waste plastic-modified asphalt pavement. Initially, the enhancement of low-temperature performance using a DVS waste plastic asphalt modifier, primarily composed of LDPE, was not highly effective for asphalt mixtures. After short-term aging, the low-temperature bending strain of the directly added waste plastic C decreased by 11.11%, and the modulus of strength increased by 14.73%; the low-temperature bending strain of D decreased by 8.12%, and the modulus of strength increased by 14.42%. Comparatively, after long-term aging, the low-temperature bending strain of C decreased by 36.19%, and the modulus of strength increased by 36.19%, while the bending strain of D decreased by 33.43% and the modulus of strength increased by 33.42%. In conclusion, it is recommended that directly added waste plastic-modified asphalt pavements should be applied in winter temperatures and in cold regions or should be compounded with other modifiers that improve the low-temperature performance of asphalt mixtures.

## 4. Environmental Protection Benefits

There are three main road polymer asphalt modifiers, as follows: thermoplastic resins such as PE, EVA, etc.; rubber such as SBR, CR, waste rubber powder, etc.; and thermoplastic rubber such as SBS, SIS, SEBS, etc. These common road polymer asphalt modifiers in the construction process often need to undergo high-speed shearing or colloid milling to achieve micron-level dispersion, and not only that, after shear dispersion, modified asphalt also needs to be melted at high temperatures to develop, during which mechanical stirring and heating temperature control are still required.

**Figure 16:**
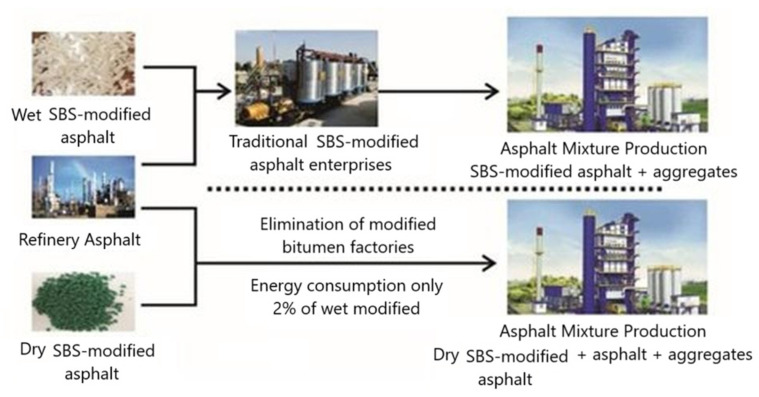
Difference between dry SBS modifiers and traditional modified asphalt.

In rubber powder materials, their melting point is very high, and the processing temperature of SBS is much higher than that of the ordinary matrix asphalt. On the one hand, this is likely to cause thermal decomposition of modifiers and asphalt aging; on the other hand, heating and temperature control often require a lot of fossil energy. In contrast, a direct injection asphalt modifier can directly skip this stage, as shown in Figure 16, mainly relying on the mixing action of the mixture in the asphalt dispersion.

Using fossil fuels to produce energy has been a major contributor to the emission of greenhouse gases (GHGs), such as carbon dioxide (CO_2_), methane (CH_4_), and nitrogen oxides (NOx). These gases trap heat in the atmosphere, leading to global warming, which in turn adversely affects the natural environment that sustains human life. The negative impacts of global warming include rising sea levels, extreme weather events, changes in biodiversity, and disruptions to agriculture, all of which threaten human societies and ecosystems worldwide. The role of fossil fuels in this environmental degradation cannot be overstated, and it is evident that industries dependent on fossil-fuel-based processes are major contributors to this problem.

One such industry is the asphalt manufacturing sector, where conventional modified asphalt processing remains heavily reliant on fossil fuels. Traditional asphalt modification methods, such as the wet process, require significant amounts of energy derived from fossil fuels to heat and blend various materials. Specifically, when compared to ordinary matrix asphalt, traditional wet-modified asphalt processing consumes approximately 10 kg of heavy oil or 7.47 kg of coal per ton of asphalt produced. This substantial fuel consumption results in significant greenhouse gas emissions, contributing directly to the ongoing climate crisis.

For each ton of modified asphalt produced using the conventional wet process, an average of 121.67 kg of carbon dioxide (CO_2_) is emitted, alongside 605.0 kg of nitrogen dioxide (NO_2_), 58.0 kg of carbon monoxide (CO), and 490.0 kg of methane (CH_4_). These emissions represent just a fraction of the total environmental footprint of traditional asphalt production. The combined impact of these gases on the atmosphere is substantial, further exacerbating the greenhouse effect and accelerating the pace of global warming.

Consider the environmental cost of paving a highway, which involves the large-scale application of modified asphalt. Suppose a two-way, four-lane highway is paved with 6 cm of fine-grained modified asphalt concrete in the upper layer and 8 cm of medium-grained modified asphalt concrete in the middle layer. For each kilometer of this highway, approximately 357.28 tons of modified asphalt would be required. This asphalt production would result in the release of 55.48 tons of carbon dioxide, 216.14 tons of nitrogen dioxide, 20.75 tons of carbon monoxide, and 175.06 tons of methane per kilometer of paved road.

Now, if a highway project spanning 150 km were constructed using conventional modified asphalt, the environmental impact would be significant. Specifically, the total carbon dioxide emissions alone would amount to 8322 tons. When accounting for all the GHGs produced (nitrogen dioxide, carbon monoxide, and methane), the overall environmental impact would be even more considerable, highlighting the substantial carbon footprint of conventional asphalt paving.

However, there is a promising alternative: using directly added (Domestic and Volatile Solid) waste plastics to modify asphalt. This innovative approach not only helps to reduce the reliance on fossil fuels in asphalt production but also repurposes waste materials, contributing to waste management and reducing plastic pollution. The environmental benefits of directly added waste plastic-modified asphalt pavements are immense. By utilizing this approach in place of conventional wet-modified asphalt, the carbon emissions associated with highway construction can be significantly reduced. The use of directly added waste plastic-modified asphalt for the same 150 km highway would result in a reduction of approximately 8322 tons of carbon emissions, demonstrating the potential for substantial environmental savings in infrastructure projects.

The adoption of waste plastic-modified asphalt could therefore play a critical role in mitigating the negative environmental impacts of traditional asphalt production. This approach aligns with global efforts to reduce carbon emissions, combat climate change, and promote sustainability in infrastructure development. The environmental benefits, coupled with the potential to reduce plastic waste, make directly added waste plastic-modified asphalt an attractive and forward-thinking solution for the future of road construction.

## 5. Conclusions

(1) High-flow polyethylene is an efficient melting and dispersing agent. High-flow polyethylene exhibits excellent compatibility with waste LDPE, functioning as a highly effective melting and dispersing agent. Its superior modification ability ensures that waste plastics can be rapidly melted and homogeneously dispersed within the asphalt mixture. This characteristic not only enhances the overall processing efficiency but also contributes to improved uniformity and consistency in the modified asphalt, which is critical for large-scale industrial applications. The improved dispersion of waste plastics also aids in optimizing the mechanical properties of the asphalt, reinforcing its structural integrity.

(2) Directional waste plastic-modified asphalt outperforms conventional modification methods. Compared to the conventional waste plastic direct spray method, directional waste plastic-modified asphalt demonstrates a significantly superior modification effect. It enhances the high-temperature performance of asphalt mixtures by increasing their resistance to rutting and deformation, making it particularly suitable for regions experiencing high traffic loads and elevated temperatures. Additionally, the overall stability of the modified asphalt is significantly improved, resulting in better durability and longer service life. This advancement presents a promising alternative to traditional asphalt modification techniques, offering a more effective and sustainable solution for infrastructure development.

(3) There are limitations in its low-temperature performance and aging effects. Despite its advantages in high-temperature conditions, directional waste plastic-modified asphalt does not provide notable improvements in low-temperature conditions. In fact, after aging, the low-temperature performance further deteriorates, indicating a potential vulnerability in cold environments. This suggests that while the material is effective in enhancing durability under heat stress, it may become brittle or more susceptible to cracking in colder climates. Therefore, it is recommended that the application of directional waste plastic-modified asphalt be primarily limited to regions with moderate to warm winters, where its high-temperature stability can be fully leveraged without compromising its long-term performance.

(4) Environmental benefits and sustainability impacts. The implementation of directional waste plastic-modified asphalt offers substantial environmental benefits. The use of this modification technique facilitates the centralized processing and consumption of large volumes of waste plastic, reducing the environmental burden associated with plastic waste accumulation. Furthermore, the preparation process significantly lowers greenhouse gas emissions compared to conventional asphalt modification methods, making it a more sustainable and eco-friendly option. By integrating waste plastics into road construction, this approach contributes to circular economy initiatives and promotes the responsible reuse of non-biodegradable materials, thereby aligning with global sustainability goals.

(5) This study highlights the potential of directional waste plastic-modified asphalt as an innovative and sustainable solution for modern road construction. The findings underscore the importance of adopting high-flow polyethylene to improve the dispersion and integration of waste plastics in asphalt, offering enhanced high-temperature performance and overall material stability. While limitations in low-temperature performance remain a concern, the strategic application in warmer climates can maximize the benefits of this technology. Moreover, the environmental advantages further reinforce the value of this approach, positioning it as a viable alternative for reducing plastic waste while simultaneously improving asphalt properties.

## 6. Future Outlook

Although this study demonstrates that waste LDPE-modified asphalt significantly improves high-temperature stability and low-temperature crack resistance, there are still many challenges that need to be further explored. Future research should focus on optimizing the dosage and modification process of waste LDPE to enhance its overall performance under extreme climate conditions. Additionally, the long-term durability, anti-aging properties, and real-world performance of waste plastic-modified asphalt still require more field validation and long-term monitoring.

Furthermore, with the growing awareness of environmental protection, the recycling of waste plastics will become an important direction for promoting the sustainability of road engineering. Future studies can explore composite modification of waste LDPE with other eco-friendly materials to further enhance the overall performance of asphalt while reducing the dependence on traditional petroleum-based materials. As technology advances, new modifier materials and more efficient processing methods may further improve the performance of asphalt and lower production costs, leading to broader applications.

In summary, waste-LDPE-modified asphalt has significant potential in improving road engineering performance, extending service life, and promoting environmental protection and resource recycling. It is worth further exploration in future research regarding its practical applications.

## Figures and Tables

**Table 1:** Main parameters of quick-melting dispersant.

Parameters	Grade	Melt Index (g/10 min)	Density (g/cm^3^)	Tensile Strength (MPa)	Elongation at Break (%)
LDPE	1l80A	50.3	0.9170	8.3	159

**Table 2:** Molecular weights of various plastics [13].

Parameters	Number Average	Weight Average	Z Average	Z + 1 Average	Viscosity Average	Peak	Distribution Width
Plastic B	320.69	137,273	392,863	839,685	106,582	84,610	32,069
Plastic C	218.09	100,581	4,166,886	1,517,427	78,839	61,158	21,809
Plastic Y	10,349	43,684	99,591	178,008	38,828	30,458	10,349

**Table 3:** LDPE recycled materials.

Grade	Ash Content (%)	Melt Index (g/10 min)	Number of Sieve (mesh)	Density (g/cm^3^)	Tensile Strength (MPa)	Elongation at Break (%)	Flexural Strength (MPa)
BLGE0201	0.50	1.04	100	0.93	13.47	909.51	4.61

**Table 4:** Aggregate composition.

Aggregate	Mass Percentage % Through the Following Sieve Holes (mm)									
	16	13.2	9.5	4.75	2.36	1.18	0.6	0.3	0.15	0.075
AC-13	100	95	80	52	33.5	21	14.5	11	7.5	5.0

**Table 5:** Rutting experiment conditions.

Experimental Temperature	Test Wheel Pressure	Experimental Wheel Travel Distance	Round-Trip Crushing Speed	Measured Range of Displacement Sensor	Measurement Accuracy
60 ± 1 °C	0.7 ± 0.05 MPa	230 ± 10 mm	42 ± 1 times/min	0~130 mm	±0.01 mm

**Table 6:** Trabecular three-point bending experiment.

Loading Mode	Experimental Temperature	Loading Rate	Fulcrum Spacing	Test Index	Index Requirement
Three-point bending	−10 ± 0.5 °C	50 mm/min	200 mm ± 0.5 mm	Maximum bending strain value during low-temperature bending test failure	More than 2000 με in cold winter areas, more than 2300 με in cold areas, and more than 2600 με in severely cold areas

**Table 7:** Marshall stability test results.

Type	Ordinary B		Directed						Instant Dispersant	
			C		D		E		Y	
Dosage (%)	Stability (kN)	FL (mm)	Stability (kN)	FL (mm)	Stability (kN)	FL (mm)	Stability (kN)	FL (mm)	Stability (kN)	FL (mm)
0	9.72	2.5	9.72	2.5	9.72	2.5	9.72	2.5	9.72	2.5
6	10.28	2.4	11.26	2.5	11.37	2.5	11.34	2.5	11.11	2.1
8	10.67	2.3	12.34	2.4	12.81	2.3	12.75	2.4	12.03	2.3
10	11.02	1.9	13.71	1.9	13.68	2.0	13.44	2.1	12.96	2.1

**Table 8:** Rutting test results of DS (dynamic stability).

Type	Ordinary	Directed			Instant Dispersant
	B	C	D	E	Y
Dosage	DS	DS	DS	DS	DS
0%	1055.4	1055.4	1055.4	1055.4	1055.4
6%	2561.9	5779.8	6235.2	6122.4	5842.1
8%	3198.2	6847.8	7463.7	7102.6	6724.3
10%	4315.1	7833.3	8426.8	8027.3	7455.9

**Table 9:** Low-temperature bending test results.

Type		Dosage (%)	Flexural Tensile Strength (MPa)	Bending Stiffness Modulus (MPa)	Maximum Bending Strain (με)
Base asphalt		0	7.32	3574.21	2048
Ordinary	B	6	8.76	4246.24	2063
		8	9.61	4151.19	2315
		10	9.63	4334.69	2217
Directed	C	6	10.53	4558.44	2310
		8	10.98	4138.71	2653
		10	11.65	4308.43	2704
	D	6	10.52	4423.88	2378
		8	10.75	4110.89	2615
		10	10.71	3921.64	2731
	E	6	10.64	4213.86	2528
		8	10.61	3698.15	2869
		10	9.71	3533.48	2748
Instant dispersant	Y	6	10.14	4492.69	2257
		8	10.23	3725.42	2746
		10	9.59	3380.33	2837

Note: The relevant data were obtained when the test specimen was damaged.

**Table 10:** Marshall stability of immersion test results.

Type		Dosage (%)	Waterlogged Stability		Residual Stability
			0.5 h	48 h	(%)
Base asphalt		0	9.72	9.01	92.70
Ordinary	B	6	10.28	9.84	95.71
		8	10.67	10.25	96.06
		10	11.37	10.95	96.31
Directed	C	6	11.26	10.97	97.42
		8	12.34	12.03	97.48
		10	13.71	13.35	97.37
	D	6	11.37	11.04	97.10
		8	12.81	12.47	97.34
		10	13.68	13.46	98.39
	E	6	11.34	10.92	96.29
		8	12.75	12.36	96.94
		10	13.44	13.14	97.77
Instant dispersant	Y	6	11.11	10.73	96.56
		8	12.03	11.74	97.59
		10	12.94	12.72	98.30

**Table 11:** Aging properties of directly added waste-plastic-modified asphalt C/D under 8% dosage.

Test Items	Type of Asphalt	Unaged	Short-Term Aging	Long-Term Aging
Marshall stability (kN)	C	12.34	13.03	13.41
	D	12.81	12.89	13.21
Dynamic stability (times/mm)	C	5779.8	7231.6	9403.5
	D	6235.2	7450.3	9779.3
Maximum flexural strain (με)	C	2653	2358	1693
	D	2615	2403	1741
Bending stiffness modulus (MPa)	C	4138.71	4503.82	4748.18
	D	4110.89	4581.77	4803.58

## Data Availability

The original contributions presented in this study are included in the article. Further inquiries can be directed to the corresponding authors.
